# Identification of t(1;19)(q12;p13) and ploidy changes in an ependymosarcoma: a cytogenetic evaluation 

**DOI:** 10.5414/NP300451

**Published:** 2012-03-05

**Authors:** Abeer Z. Tabbarah, Austin W. Carlson, Angelica Oviedo, Rhett P. Ketterling, Fausto J. Rodriguez

**Affiliations:** 1 Department of Pathology, George Washington University, Washington, D.C.,; 2 Department of Laboratory Medicine and Pathology, Mayo Clinic, Rochester, MN, USA,; 3 Children’s and Women’s Health Centre of BC Anatomic Pathology, Vancouver, British Columbia, Canada and; 4 Department of Pathology, Johns Hopkins University, Baltimore, MD, USA

**Keywords:** Key words

## Abstract

Gliosarcoma, a recognized subtype of glioblastoma, is a biphasic tumor exhibiting distinct glial and sarcomatous components. Ependymosarcomas are rarer, biphasic ependymal tumors exhibiting sarcomatous change. Genetic abnormalities associated with this curious phenotype are not well understood. We are presenting the first karyotype of ependymosarcoma with identification of a clonal t(1;19)(q12;p13). Fluorescence in situ hybridization (FISH) was performed with a probe set targeting 1q23 and 19p13.3. Although the tumor did not show evidence of t(1;19)(q23;p13.3) by FISH, increased ploidy was a feature of the sarcomatous component. On clinical follow-up the patient is doing well without evidence of recurrence 55 months after initial resection, and postoperative treatment with irradiation and temozolomide. The significance of the genetic alterations we describe associated with sarcomatoid change in ependymal neoplasms, and ultimately their prognostic relevance, merits further study.

## Introduction 

Gliosarcoma, a variant of glioblastoma, is a biphasic malignant glial tumor composed of a glial and a sarcomatous component. In most cases, the glial component is astrocytic [5]. However, there have been reports of the glial component being oligodendroglial [17] or ependymal [2, 9, 10, 18, 21]. The terms oligosarcoma and ependymosarcoma have been proposed for such composite tumors [17, 18]. 

Given the rarity of these tumors, genetic characterization and elucidation of specific alterations underlying their development are lacking in the literature. In this report we present conventional cytogenetic findings of an ependymosarcoma case, along with fluorescence in situ hybridization (FISH) analysis for ploidy. 

## Materials and methods 

### Case history

**Figure 1:**
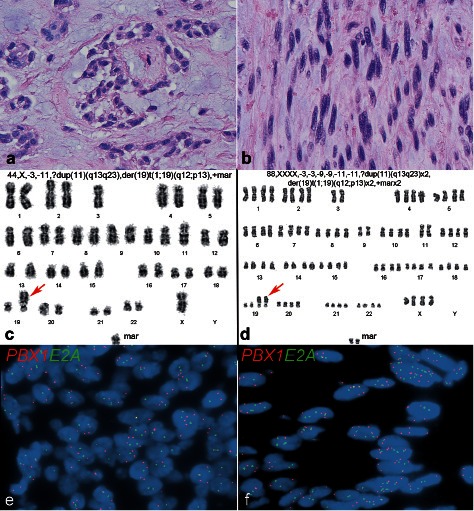
Increased ploidy in sarcomatous component of ependymosarcoma. An ependymosarcoma with distinct ependymal (a) and sarcomatous (b) components was subjected to cytogenetic analysis. Conventional cytogenetics revealed several clonal abnormalities, including a t(1;19)(q12;p13) translocation (arrow) (c). In addition a subset of metaphases demonstrated tetraploidy (d). Dual color FISH studies performed in the ependymal (e) and sarcomatous (f) components demonstrated a lack of *PBX1/E2A* fusion, but increased ploidy in the sarcomatous component.

Clinical and pathologic features of this tumor have been previously described [18]. Briefly, the patient was a 13-year-old girl who presented with new onset seizures. Magnetic resonance imaging revealed an enhancing calcified mass with surrounding edema in the right frontal lobe. Gross total resection revealed a firm, nodular, well demarcated, focally infiltrating mass. Postoperatively, the patient underwent radiation therapy and received temozolomide. Microscopic examination revealed an ependymosarcoma composed of focal myxopapillary-like ependymal areas and a sarcomatous component resembling fibrosarcoma (Figure 1a, b). Immunohistochemical stains supported the morphologic impression [18]. On updated clinical follow-up, the patient is alive and without evidence of recurrent disease during her last clinic visit, 55 months after resection. Her seizures are well controlled by medication. 

### Cytogenetic analysis

Metaphase chromosomes were prepared and G-banded for karyotyping using standard methods on cultured cells obtained from fresh tumor tissue. Dual fluorescence FISH was performed using probes designed from bacterial artificial chromosomes (BAC) targeting *PBX1* (1q23) and *E2A* (e.g., *TCF3* at 19p13.3). The *PBX1* and *E2A* BACs were labeled with Spectrum Orange-dUTP^TM^ (Vysis Inc., Downers Grove, IL, USA) and Spectrum Green-dUTP^TM^ (Vysis Inc.), respectively, as previously reported [19]. Only FISH evaluation was performed separately in the ependymal and sarcomatous components. 

## Results 

Conventional cytogenetic analysis demonstrated the following clonal abnormalities: 44,X,-3,-9,-11,?dup(11)(q13q23),der(19)t(1;19)(q12;p13),+mar (Figure 1c). A subset of metaphases demonstrated a tetraploid subclone with the same abnormalities (Figure 1d). FISH analyses using probes designed to identify *PBX1*/*E2A* fusion associated with t(1;19)(q23;p13.3) in precursor B-cell ALL were performed, since it was the closest probe set available to evaluate t(1;19). There was no evidence of *PBX1*/*E2A* fusion. However, the glial component showed 2-4 *PBX1* (red) and 1-2 *E2A* (green) FISH signals (75% of cells) (Figure 1e), while the mesenchymal areas had more of a range of FISH signals, with ~ 40% of nuclei demonstrating 5 – 6 *PBX1* and 4 *E2A* signals (Figure 1f). These signal patterns were consistent with the karyotypic findings, supporting that the glial component was diploid and the sarcomatous component more tetraploid, although cells for conventional cytogenetics were not separated prior to analysis. 

## Discussion 

Ependymosarcomas are rare tumors, with less than 20 cases reported in the literature [21]. Reports include 2 cases of subependymoma with sarcomatous change [11, 12, 18], 1 case of mixed subependymoma-rhabdomyosarcoma [20], and 15 cases of sarcomatous change in ependymoma [7, 9, 10, 18, 21]. The ependymomas described have been classified as WHO Grade II or Grade III [2, 9, 10, 17, 21]. Seven of the 15 ependymosarcomas were described in the original resection specimens [7, 18], while 8 were described in recurrent tumor specimens [2, 9, 10, 18, 21]. Seven of the 15 cases were described after the patient had received radiation therapy [2, 9, 10, 18]. Whether these tumors are radiation induced depends on a number of factors, including defining the time interval required between the radiation and the development of the sarcomatous component [18]. 

Multiple studies over the years have attempted to elucidate the pathogenesis of gliosarcomas. Initially, the glial and mesenchymal components were thought to arise from different cells of origin as per the “polyclonal hypothesis”. The mesenchymal element was thought to develop from fibroblasts, pluripotent cells of the vascular adventitia or perivascular spaces, vascular smooth muscle cells, or monohistiocytic cells [1]. According to the “monoclonal hypothesis”, the mesenchymal component develops from glial precursors during tumor progression [1]. More recent molecular genetic studies have shown that both glial and mesenchymal components share common genetic aberrations in most instances, therefore supporting the monoclonal hypothesis [1, 3, 4, 16, 18]. Chromosomal imbalances identified in gliosarcomas include gains on chromosomes 7, X, 9q, 12q, and 20q, and losses on chromosomes 10, 9p, 13q, and 17 [1, 4, 8]. Genetic imbalances are similar in both glial and sarcomatous components in most cases studied. 

It is of interest that molecular cytogenetic study of this example of ependymosarcoma demonstrated increased ploidy changes in the sarcomatous component. The sarcomatous component of this tumor also had polysomies of chromosomes 11 and 12 [18]. In addition, another case of ependymosarcoma (of 4 cases tested) in the series by Rodriguez et al. (Case 2) also displayed polysomies of chromosomes 11 and 12 restricted to the sarcomatous component. This combination of findings is intriguing, and raises the possibility that ploidy changes may in part explain the development of this unique morphologic variation at the genetic level. 

Rearrangements involving chromosomes 1 and 19 have been reported in multiple cancers [14]. A t(1;19)(q12;p13) has been detected in 3 cases of malignant melanoma [15]. The breakpoint in chromosome 19 appeared similar to that reported in precursor B-cell leukemia, and at the time the human insulin receptor gene, grossly mapping to this region, was felt to be the involved. However, subsequently it was discovered that precursor B-cell acute lymphoblastic leukemia has a *PBX1*/*E2A* fusion gene characterized by either a der(19)t(1;19)(q23;p13.3) or t(1;19)(q23;p13.3).The probe set designed to identify these translocations [19] was used in the FISH analysis of our case. A t(1;19)(q23;q13) has also been reported in a medulloblastom aand glioblastoma [22]. Another case involving 19p13 is seen in t(1;19)(p22;p13.1), identified for the first time in a mixed epithelial stromal tumor (MEST) of the kidney [6]. Not unlike ependymosarcomas, MEST are biphasic tumors composed of epithelial and mesenchymal elements. Lui et al. [13] reported similar cytogenetic findings in a highly malignant primitive soft tissue sarcoma, suggestive of a rhabdomyosarcoma variant, with a hypertetraploid karyotype and t(1;19)(q12;q13.2). The increased ploidy and t(1;19) involving 1q12 are similar to the findings we report in the sarcomatous component in our case, although detailed mapping is lacking. 

The relationship of these genetic alterations with prognosis is unclear at the present time. Sarcomatoid change occurring in tumors outside the nervous system portends more aggressive behavior. The prognosis in the prior ependyomosarcoma series was variable, not uniformly dismal [18], and the patient in this report remains disease free 55 months after resection. The genetic alterations described in this case of ependymosarcoma are intriguing. Future studies should be of value in clarifying their clinical and/or biological significance, and identifying the specific genes involved in these alterations.
